# A domain key-based secure SOME/IP protocol

**DOI:** 10.1371/journal.pone.0331069

**Published:** 2025-09-05

**Authors:** Jaewoong Heo, Hyunghoon Kim, Hyojin Jo

**Affiliations:** 1 School of Software, Soongsil University, Seoul, South Korea; 2 Graduate School of Information, Yonsei University, Seoul, South Korea; King Fahd University of Petroleum & Minerals, SAUDI ARABIA

## Abstract

With the introduction of Advanced Driver Assistance Systems (ADAS), modern vehicles are equipped with numerous sensors, significantly increasing data communication within the in-vehicle network. However, the limited bandwidth of the Controller Area Network (CAN) poses challenges for high-speed sensor data transmission. To address this, automotive ethernet is emerging as a replacement for CAN, enabling the efficient transmission of large volumes of data, such as from cameras and LiDAR. Supporting this transition, SOME/IP (Scalable Service-Oriented Middleware over IP) has been introduced as middleware to enable service-oriented communication through Request/Response and Publish/Subscribe mechanisms. Despite its advantages, SOME/IP lacks essential security measures, such as authentication and encryption, making it vulnerable to attacks, including man-in-the-middle attack scenarios where attackers inject fake SOME/IP messages. However, existing security approaches, such as group key-based protocols and pairwise key-based methods utilizing authentication servers, present limitations. Group key-based methods are not secure against node compromise attacks, while pairwise key-based approaches face single point of failure issues due to their reliance on authentication servers.

To address these challenges, this paper proposes a domain key-based secure SOME/IP protocol. By leveraging domain keys, the proposed approach limits the impact of node compromise attacks to the specific domain of the compromised node, while eliminating the single point of failure problem. Experimental results demonstrate that the session establishment time increases by only 5–10 ms, and the message transmission time increases by up to 115 ms compared to the existing group key-based SOME/IP protocol.

## 1 Introduction

With advances in IT technology, various innovations are being integrated into modern vehicles, accelerating their digitization. The application of IT in vehicles not only boosts fuel efficiency but also significantly enhances driver safety and convenience. As a result, systems such as Lane Keeping Assist Systems (LKAS) and Advanced Driver Assistance Systems (ADAS) are increasingly adopted to provide additional safety and ease of use for drivers [1,2].

To ensure precise control over these systems, numerous Electronic Control Units (ECUs) and sensors are embedded within vehicles [3], leading to a substantial increase in communication traffic within the vehicle’s internal network [4]. Currently, the primary in-vehicle network, the Controller Area Network (CAN), operates with a maximum bandwidth of 1 Mbps and a data payload limit of 8 bytes per message [5].

However, limited bandwidth and data capacity of CAN restrict its effectiveness for high-demand services, such as camera-based systems, that require real-time, large-scale data exchange. To address these limitations, automotive ethernet is being increasingly adopted in vehicles [6,7]. This standard includes 100Base-T1 (IEEE 802.3bw) and 1000Base-T1 (IEEE 802.3bp), both of which support a maximum MTU of 1500 bytes. While 100Base-T1 offers speeds of up to 100 Mbps, 1000Base-T1 supports speeds of up to 1 Gbps.

The integration of automotive ethernet has further facilitated the adoption of Service-Oriented Architecture (SOA) for efficient communication among ECUs [8]. To support this advancement, the automotive industry consortium, AUTOSAR, has adopted Scalable service-Oriented Middleware over IP (SOME/IP) as the middleware standard for SOA-based in-vehicle communication. SOME/IP provides several key advantages for in-vehicle networks, including:

- Flexibility in Communication: SOME/IP supports various types of messaging, such as publish-and-subscribe, request-response, and event notifications, allowing ECUs to communicate in real-time based on different application requirements.
- Multicast Support: By facilitating multicast communication, SOME/IP minimizes network load when delivering identical messages to multiple ECUs.
- Interoperability: The middleware enables seamless communication across diverse platforms and networks, ensuring compatibility with other ethernet-based technologies and enhancing system integration capabilities.

Despite its benefits, SOME/IP lacks built-in security features, making it vulnerable to ethernet-based network attacks [9]. To address this, a certificate-based session establishment protocol has been proposed [10]. In this protocol, each ECU uses its own certificate to verify permission to publish or subscribe to services. Upon successful verification, a group key-based secure session is established between a server ECU and a client ECU. However, since all client ECUs receiving messages from a server ECU share the same group key, compromising a single client ECU enables it to send spoofed messages to other client ECUs. This implies that the security of all sessions between the server ECU and its clients is jeopardized by the compromise of just one ECU. Similarly, the SESO-RC protocol proposed in [11] also relies on a group key for message protection, allowing any compromised client ECU to publish unauthorized messages to other nodes in the network.

Another approach involves central node-based solutions, such as the SESO-RC protocol [11] and an authentication ticket-based protocol [12], where a server ECU and each client share a unique key. While this method improves security by eliminating the use of a shared group key, it introduces significant challenges. First, it creates a single point of failure, as communication is entirely disrupted if the central node becomes unavailable. Furthermore, the server ECU must individually encrypt and authenticate messages for each client before transmitting them via unicast, leading to considerable communication and computational overhead.

To address the limitations of previous studies, this paper proposes a domain key-based secure SOME/IP protocol. In this method, a message publisher ECU generates a unique domain key for each domain (e.g., body domain, powertrain domain, infotainment domain, etc.) in the vehicle. The generated domain session key is then shared exclusively with the ECUs within the corresponding domain. With this approach, even if an attacker obtains a domain session key from a compromised ECU, the impact is confined to the domain to which the compromised ECU belongs, preventing any adverse effects on communications in other domains. The contributions of this paper are as follows:

- A domain key-based security protocol for SOME/IP is proposed. This protocol introduces a new domain certificate that includes a service identifier and a domain identifier. This allows a server ECU to verify whether its service is accessible by a specific client ECU and facilitates the management of distinct domain session keys for each domain. As a result, even if a client ECU is compromised and its credentials are leaked, the impact of the attack is restricted to the domain of the compromised ECU.
- Through experiments, it was confirmed that the session establishment time increased by 5–10 μs and the message transmission time increased by up to 115 μs compared to the existing group key-based secure SOME/IP protocol. It was demonstrated that the increase in overhead is not significant when compared to the benefit of restricting the attack scope.

The structure of this paper is as follows: Sect 2 explains the related research, and Sect 3 describes the background knowledge. Sect 4 introduces the protocol proposed in this paper. Sect 5 presents the experiments conducted to evaluate the performance of the proposed protocol and compares it with existing studies. Sect 6 discusses the limitations of this paper and potential directions for future research. Finally, Sect 7 concludes the paper.

## 2 Related works

The study by Iorio et al. [10] introduces a novel security protocol for SOME/IP, defining three distinct security levels: no security (nosec), authentication, and encryption. Secure sessions are established through certificate-based authentication between a server ECU and a client ECU. In this protocol, certificates incorporate SOME/IP-specific details, including the service ID, instance ID, offer/request permissions, and security levels. During session establishment, the server ECU validates the client ECU’s certificate, which is embedded in the service request message, to ensure the client is authorized to subscribe to the service. If authorization is granted, the server ECU generates a random group key and encrypts it using the client ECU’s public key. The server’s response to the service request contains its certificate, the selected security level, the encrypted group key, and a digital signature on the response. Upon receiving the response, the client ECU verifies the server ECU’s digital signature. If the digital signature is valid, the client ECU decrypts the group key using its private key. Thereafter, communication between the server and client ECUs is encrypted and authenticated using the shared group key. While this protocol introduces additional overhead due to the group key establishment and encryption/decryption processes, it effectively mitigates threats such as forgery, tampering, and injection of SOME/IP messages by unauthorized attackers without access to the group key. However, a notable limitation remains: if an ECU is compromised, the group keys for all services it subscribes to may be exposed, potentially enabling spoofing attacks on those services.

Zelle et al. [11] investigated multiple Man-In-The-Middle (MITM) attack scenarios within the SOME/IP protocol and demonstrated the practicality of these attacks through detailed procedures. To counter such vulnerabilities, they introduced two security enhancements. The first, SESO-RC, utilized a certificate-based mutual authentication approach. In the process of exchanging messages via the SOME/IP protocol, the Diffie-Hellman (DH) key exchange was employed to generate session keys. Specifically, for Publish/Subscribe scenarios, SESO-RC adopted a group key distribution mechanism. In this approach, the server ECU creates a group key, encrypts it individually for each client using the client’s session key, and then distributes it to all client ECUs. The second enhancement, SESO-AS, incorporates an authentication server to strengthen security. This method relies on long-term keys and pre-configured policies for each ECU within the authentication server. When the server ECU sends a service offer message to the authentication server, the authentication server first validates the message using a pre-shared key established between the server ECU and the authentication server, followed by a policy check. After validation, the authentication server facilitates the distribution of the service offer messages to client ECUs authorized to access the service. During this process, the session key for each client ECU is encrypted using the long-term key shared between the authentication server and the respective client ECU, ensuring secure delivery. However, the two protocols, SESO-RC and SESO-AS, have the following limitations. First, SESO-RC is vulnerable to node compromise attacks. If a node is compromised and the group key for a specific service is leaked, messages associated with that service can be manipulated. On the other hand, SESO-AS suffers from a single point of failure issue on the authentication server, which poses significant risks to system availability.

A study similar to SESO-AS has been proposed in [12]. This method incorporates mutual server-client authentication and service policy verification through an authentication server. Authentication tickets issued by the authentication server ensure secure services between a server ECU and client ECUs. However, in this study, the authentication server also faces a single point of failure, which could lead to critical availability issues. Moreover, the reliance on pairwise keys between the server and clients introduces inefficiencies, particularly in publish-and-subscribe-based multicast services. In such cases, the server ECU must perform encryption and authentication operations individually for each client ECU receiving the service, resulting in substantial overhead and diminished scalability.

## 3 Background

### 3.1 SOME/IP

Scalable service-Oriented Middleware over IP (SOME/IP) is a communication middleware standardized by the AUTOSAR consortium to enable modern, flexible, and efficient on-board architectures in automotive systems [13]. By adopting a service-oriented architecture, SOME/IP abstracts low-level network details, such as IP addresses and ports, allowing applications to interact through well-defined interfaces that represent specific services. The architecture supports multiple instances of the same service, uniquely identified by instance IDs, which can exist across different ECUs or within a single device.

**Fig 1:**
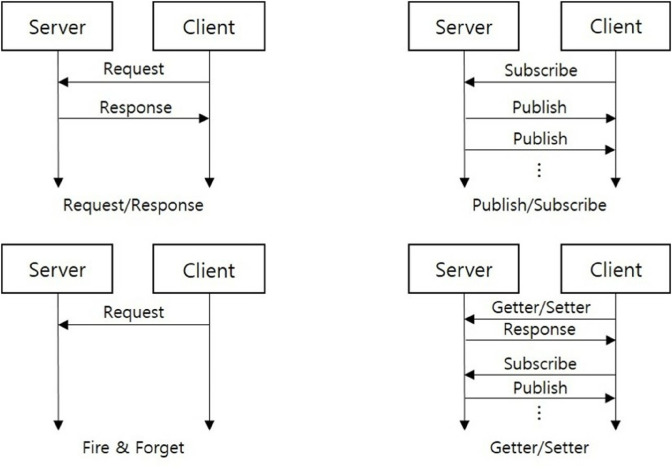
SOME/IP protocols.

SOME/IP includes a service discovery mechanism (SOME/IP-SD) that facilitates the dynamic identification and interaction between service providers and consumers. When a client needs to access a service, SOME/IP-SD enables server-client communication using the method illustrated in Fig 1. The communication models provided by SOME/IP are diverse. The Request/Response model, similar to Remote Procedure Calls (RPC), enables clients to invoke functions on remote ECUs and receive corresponding results [14]. This model supports unicast communication. The Publish/Subscribe model follows an event-driven approach, where the server sends messages to clients subscribed to its service. Supporting both unicast and multicast communication, this model allows efficient message distribution to multiple clients subscribed to specific topics. Fire-and-Forget communication model occurs when a client sends a request message, but the server does not return a response. Finally, the Getter/Setter model is used for state management, allowing clients to retrieve values using Getter messages or modify them using Setter messages. Additionally, when a state value changes or reaches a specific interval, the server can publish the updated value to the client.

SOME/IP operates on transport protocols such as UDP and TCP, accommodating varying requirements for latency, reliability, and data size. UDP, with its low overhead and multicast support, is ideal for scenarios requiring minimal latency and efficient network utilization. TCP, on the other hand, is suitable for transferring large data chunks where reliability is critical but latency constraints are less stringent. SOME/IP has become a foundational component of modern automotive ethernet networks, gradually replacing traditional technologies such as CAN bus and FlexRay. With its support for service-oriented communication, dynamic service discovery, and robust transport models, SOME/IP plays a crucial role in enabling scalable and high-performance architectures for next-generation vehicles.

However, SOME/IP lacks native security mechanisms, such as authentication, integrity, and confidentiality, delegating these responsibilities to the transport layer. This reliance can introduce additional overhead and limit flexibility in some implementations. To mitigate these issues, supplementary security protocols such as AUTOSAR SecOC or MACsec are often integrated, ensuring message authenticity and protecting the network from unauthorized access or malicious attacks.

### 3.2 SOME/IP Header

**Fig 2:**
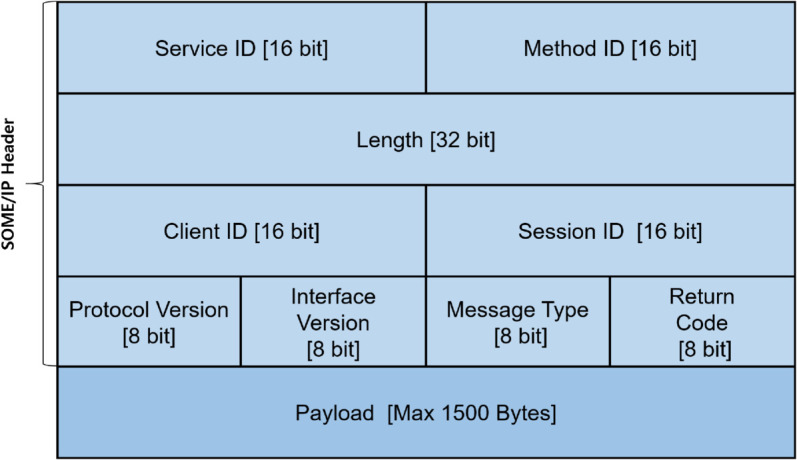
The packet structure of SOME/IP.

The header structure of SOME/IP, shown in Fig 2, is composed of the following fields:

- Service ID: Identifies the service provided by the server.
- Method ID: Differentiates between methods and events. Values from 0 to 32767 represent methods, while 32768 to 65535 indicate event communication.
- Length: Specifies the total length of the SOME/IP message, including the header and payload.
- Client ID: Identifies the client within the ECU.
- Session ID: Provides a unique identifier for each communication session.
- Protocol Version: Indicates the version of the SOME/IP protocol in use.
- Interface Version: Represents the version of the service interface being used.
- Message Type: Denotes the type of message being transmitted.
- Return Code: Indicates the result of message processing, such as success or error.
- Payload: Contains the SOME/IP message content.

### 3.3 vsomeip

vsomeip is an open-source framework that implements the SOME/IP protocol and provides a software architecture to manage communication between ECUs within a vehicle [15]. It is compatible with POSIX-compliant operating systems, enabling it to be ported across various platforms while meeting the communication requirements specified by AUTOSAR [16].

To establish communication between two ECUs using vsomeip, a JSON (JavaScript Object Notation) configuration file must be created. This file specifies essential communication parameters, such as multicast communication settings, the use of Service Discovery (SD), Service/Instance IDs, the ECU’s IP address, and port number. It serves as the primary communication configuration for vsomeip. Communication is initiated through a module called the Routing Manager, which is responsible for managing message paths. The Routing Manager supports service discovery and invocation by analyzing SOME/IP message headers to extract key details, including the destination IP address, port number, and service ID. Based on this information, the ECU establishes communication with the intended target.

## 4 Domain key-based secure SOME/IP protocol

### 4.1 Adversary model

We assume that an adversary can infiltrate the automotive ethernet through vulnerable physical or remote access interfaces present in the vehicle. The attacker may insert, replay, eavesdrop on, modify, or drop arbitrary messages on the automotive ethernet. However, Denial-of-Service (DoS) attacks are beyond the scope of this work and are left for future research. Additionally, we assume that the adversary can compromise ECUs and, through this, gain access to credentials stored within the ECUs, such as encryption and authentication keys.

Since each ECU is included in a specific domain—such as body, powertrain, chassis, or infotainment—and the vulnerable interfaces exploited by the adversary differ across domains, we further assume that compromising multiple ECUs across different domains simultaneously poses a significant challenge for adversaries. This assumption is reasonable because cyberattacks are typically initiated from the infotainment domain.

### 4.2 System overview

**Fig 3:**
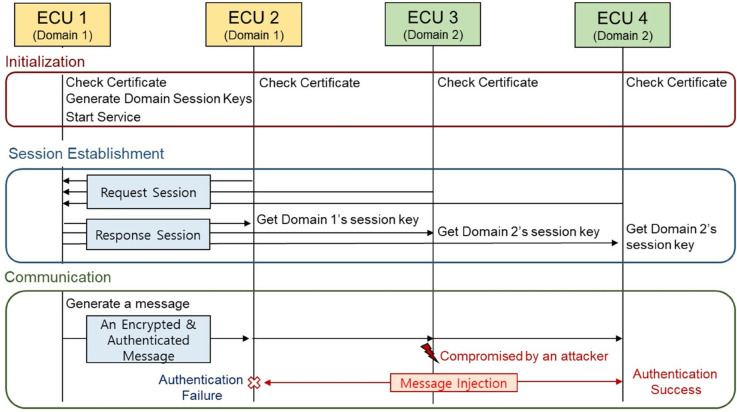
The overview of the domain key-based secure SOME/IP protocol.

The domain key-based secure SOME/IP communication security framework, illustrated in Fig 3, comprises three phases: the initialization phase, the session establishment phase, and the communication phase. In the initialization phase, domain certificates containing the domain ID and domain-specific policies for each ECU are installed. These domain IDs and policies are used to verify whether a specific domain is authorized to provide or consume services. The session establishment phase describes the process of setting up a secure session between a server ECU and a client ECU using the domain certificates. During this phase, a domain session key is exchanged to ensure secure communication. Lastly, the communication phase focuses on the secure exchange of messages between the server ECU and the client ECU, utilizing the domain session key established during the session setup. Table 1 provides an explanation of the notations used in the proposed protocol.

**Table 1:** Notations.

Notations	Description
${\text{ECU}}_{i}$	An electronic control unit *i*
${\text{Domain}}_{d_{i}}$	A domain *i*
${\text{srv}}_{i}$	A service identity provided by ${\text{ECU}}_{i}$
${\text{cert}}_{i}$	A domain certificate of ${\text{ECU}}_{i}$
${\text{n}}_{i}$	A random number generated by ${\text{ECU}}_{i}$
${\text{pk}}_{i}$	A public key of ${\text{ECU}}_{i}$
${\text{sk}}_{i}$	A private key of ${\text{ECU}}_{i}$
${\text{dsk}}_{d_{i},i}$	A domain session key for ${\text{ECU}}_{i}$ in ${\text{Domain}}_{d_{i}}$
${\text{enckey}}_{d_{i},i}$	An encrypted key for ${\text{ECU}}_{i}$ in ${\text{Domain}}_{d_{i}}$
${\text{sign}}_{i}$	A digital signature signed by ${\text{ECU}}_{i}$’s ${\text{sk}}_{i}$

### 4.3 Initialization

All ECUs within a vehicle are assumed to be part of a domain, and during the vehicle manufacturing process, each ECU is provisioned with a certificate, a public key, and a private key. Each certificate contains a policy that defines the permissions of its owner. The policy structure is based on prior research [10], with the addition of a domain ID. This domain ID is used to determine whether an ECU can provide or consume a specific service within the domain. The policy format of each independent authorization rule in the certificate has the following structure:

$$app,srv,role,min_{sl},domain$$ (1)

Here, *app* identifies the application to which the rule applies, and *srv* represents the SOME/IP service to which the rule refers. The *role* defines that the *app* can assume for the specified service instance, such as offering (i.e., implementing the interface defined by the service) or requesting (i.e., consuming the functionalities provided by the service). *min*_*sl*_ specifies the minimum security level required to use the service and is divided into two modes: nosec and sec. The nosec mode refers to a SOME/IP service without security features, while the sec mode indicates a domain key-based secure SOME/IP service. Lastly, *domain* specifies the domain identifier where the service can be utilized.

**Fig 4:**
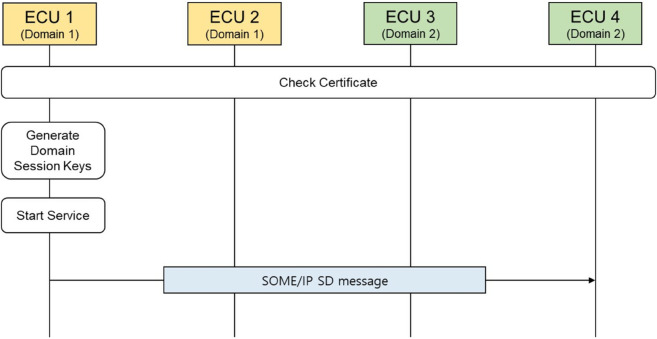
The initialization of the domain key-based secure SOME/IP protocol.

Once the certificates are installed on each ECU, a server ECU verifies, using its certificate, whether it has the authority to offer the specified service before initializing the service. If the server ECU is authorized to provide the service, it generates random domain session keys for each domain within the vehicle. These keys ensure the confidentiality and authenticity of the SOME/IP messages related to the service. Subsequently, the server ECU broadcasts the service ID through a SOME/IP-SD message to announce the availability of the service, informing other client ECUs that the service is being offered. Fig 4 shows the initialization of domain key-based secure SOME/IP protocol.

### 4.4 Session establishment

The session establishment process ensures secure communications between a server ECU and a client ECU. This process includes mutual authentication, verification of domain authorization for providing or requesting a service, and secure transport of domain session keys. The steps involved in this process are as follows.

- When a client ${\text{ECU}}_{i}$, within a specific domain (${\text{Domain}}_{d_{i}}$), receives a SOME/IP-SD message containing the desired service, it first verifies its authorization using the permissions embedded in its certificate. If ${\text{ECU}}_{i}$ has the required permissions for the SOME/IP-SD message, it proceeds to generate a session establishment request. To protect against replay attacks, ${\text{ECU}}_{i}$ generates a random nonce, ${\text{n}}_{i}$. The request includes ${\text{ECU}}_{i}$’s certificate, the nonce ${\text{n}}_{i}$, and its domain ID, ${\text{Domain}}_{d_{i}}$. This message is then digitally signed and transmitted to the server ECU.
- Upon receiving the session establishment request, the server ECU validates the message to determine if it should provide the domain session key, ${\text{dsk}}_{d_{i},i}$, for ${\text{ECU}}_{i}$ in ${\text{Domain}}_{d_{i}}$. First, the server verifies the client’s certificate to ensure the client is authorized to request the specified service and confirms that the domain ID in the certificate matches the transmitted domain ID. Once the certificate is validated, the server checks whether the minimum security level specified in the client’s certificate meets the requirements for its services. If all validations are successful, the server retrieves the domain session key ${\text{dsk}}_{d_{i},i}$ associated with ${\text{Domain}}_{d_{i}}$. This key is encrypted using the client’s public key (${\text{PK}}_{i}$), producing an encrypted domain key (${\text{enckey}}_{d_{i},i}$). The server then creates a response message, including the client’s nonce (${\text{n}}_{i}$), its own certificate, and the encrypted domain key. This response is signed with the server’s private key and sent to the client ECU.
- After receiving the response message, ${\text{ECU}}_{i}$ checks whether the server is authorized to provide the requested services. It also verifies that the nonce received from the server matches the nonce it originally sent, ensuring the response is not replayed or tampered with. Additionally, the server’s signature on the response message is validated to confirm its integrity and authenticity. Once all validations are complete, the client decrypts the encrypted domain session key (${\text{enckey}}_{d_{i},i}$) using its private key (${\text{SK}}_{i}$). This process allows the client to retrieve the domain session key ${\text{dsk}}_{d_{i},i}$, which are then used for secure communication within the domain.

**Fig 5:**
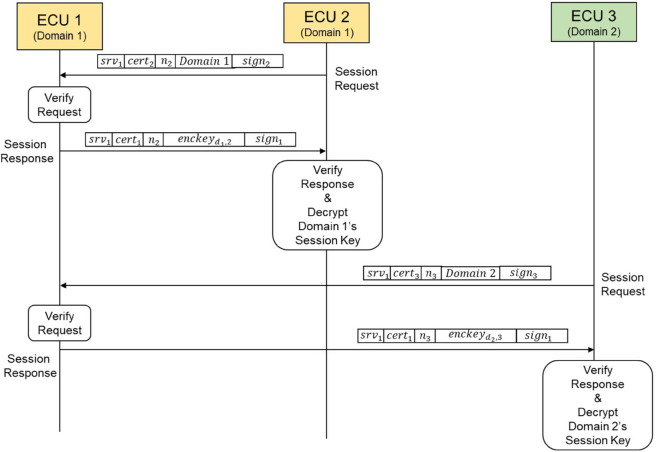
The session establishment of the domain key-based secure SOME/IP protocol.

Fig 5 provides an example of the handshake process between the server ECU and the client ECU during session establishment.

### 4.5 Communication

The process of SOME/IP communications protected by the domain key is as follows:

- First, whenever an event occurs, the server ECU encrypts the SOME/IP messages to be published for all domains and generates authentication values using the Authenticated Encryption with Associated Data (AEAD) algorithm based on each domain session key it has created. It then sets the Client ID field in the SOME/IP header to the Domain ID for each domain. Subsequently, the server ECU publishes messages to the client ECUs.
- The client ECU monitors all messages published by the server ECU and verifies whether the value in the Client ID filed of the SOME/IP header matches its own domain ID. Upon receiving a message that matches its domain ID, the client ECU verifies the integrity of the message using the authentication value included in the payload, and decrypts the encrypted SOME/IP message.

**Fig 6:**
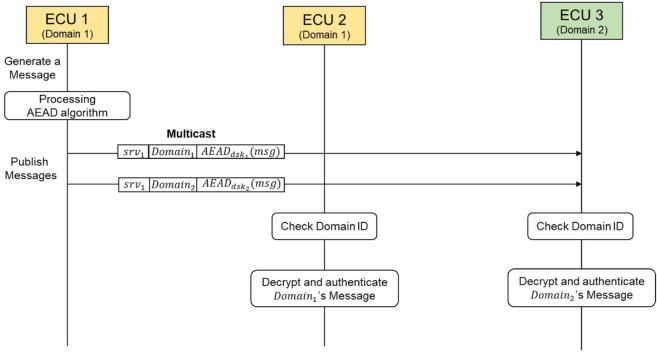
The publish/subscribe communication of the domain key-based secure SOME/IP protocol.

The communication process is illustrated in Fig 6, which depicts the publish/subscribe communication mechanism with domain key-based secure communication applied.

## 5 Evaluation

### 5.1 Security analysis

The security of the proposed domain key-based secure SOME/IP SOME/IP protocol is based on well-established cryptographic assumptions and adversarial models relevant to in-vehicle networks. A detailed security analysis is presented below.

#### 5.1.1 Session establishment security.

The session establishment phase of the protocol is designed to ensure that only an authorized client ECU, identified as ${\text{ECU}}_{i}$, can obtain the corresponding domain session key ${\text{dsk}}_{d_{i},i}$ securely provisioned by the server ECU. To achieve this, the protocol employs a standardized public key cryptographic system such as RSA for ensuring confidentiality through encryption and guaranteeing authenticity via digital signatures.

We assume that the RSA encryption scheme used is semantically secure and one-way, such that an adversary without the private key ${\text{sk}}_{i}$ cannot decrypt the ciphertext ${\text{enckey}}_{d_{i},i}$. Additionally, the signature scheme employed is assumed to be existentially unforgeable under chosen-message attacks (EUF-CMA), preventing any adversary from generating valid signatures without access to the signer’s private key.

Suppose a probabilistic polynomial-time adversary 𝒜 attempts to compromise the domain session key of a non-compromised ECU. In that case, 𝒜 must either (i) forge a valid certificate or signature, (ii) break the RSA encryption to recover ${\text{dsk}}_{d_{i},i}$, or (iii) replay an earlier response message with a stale nonce ${\text{n}}_{i}$. Each of these attacks contradicts our security assumptions. Case (i) would imply a successful forgery under EUF-CMA, case (ii) would contradict RSA’s one-wayness, and case (iii) is ruled out by the use of fresh, random nonces and strict validation procedures. Therefore, we conclude that the probability that 𝒜 successfully learns ${\text{dsk}}_{d_{i},i}$ is negligible in the security parameter, i.e.,


$$\mathrm{Pr}\left[{𝒜}_{\text{RSA}}\text{ learns }{\text{dsk}}_{d_{i},i}\right]\leq \varepsilon_{\text{RSA}}(n_{\text{RSA}}),$$

where $\varepsilon_{\text{RSA}}(n_{\text{RSA}})$ is a negligible function in the RSA security parameter $n_{\text{RSA}}$.

#### 5.1.2 Communication phase security.

In the communication phase, the protocol ensures that messages transmitted between a server ECU and authorized client ECUs are both confidential and authentic. This is achieved through the use of authenticated encryption with associated data (AEAD), such as AES-GCM, which simultaneously provides both encryption and message authentication.

We assume that the AEAD scheme is secure against chosen-ciphertext attacks (IND-CCA) and provides strong integrity guarantees. That is, any adversary attempting to forge a valid AEAD ciphertext or distinguish encrypted messages without the session key should only succeed with negligible probability. Let us consider an adversary 𝒜 who does not possess ${\text{dsk}}_{d_{i},i}$ but attempts to either decrypt an intercepted ciphertext or forge a new valid ciphertext. Any such attempt would imply either a breach of confidentiality or integrity of the AEAD scheme, which contradicts our cryptographic assumptions. Hence,


$$\mathrm{Pr}\left[{𝒜}_{\text{AEAD}}\text{ forges or decrypts AEAD ciphertext}\right]\leq \varepsilon_{\text{AEAD}}(n_{\text{AEAD}}),$$

where $\varepsilon_{\text{AEAD}}(n_{\text{AEAD}})$ is a negligible function in the AEAD security parameter $n_{\text{AEAD}}$.

As a result, all AEAD-protected messages in the communication phase remain secure under standard adversarial models. The adversary’s advantage in violating either the confidentiality or integrity of these messages is bounded by a negligible function in the security parameter.

### 5.2 Performance analysis

#### 5.2.1 Experimental setup.

**Fig 7:**
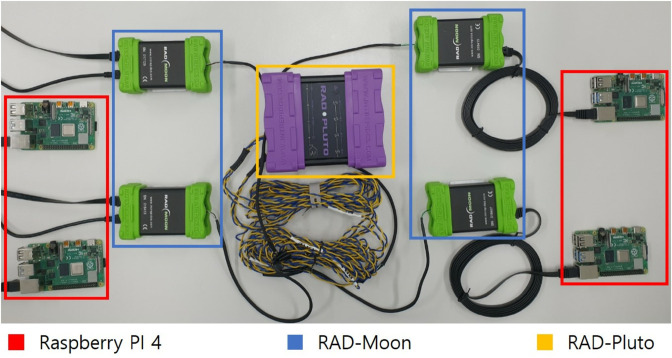
The experimental setup.

To evaluate the performance of the proposed protocol, the experiments were conducted using Raspberry Pi 4 (@ 1.5 GHz), RAD-Moon, and RAD-Pluto, with the experimental setup configured as shown in Fig 7. In this setup, four Raspberry Pi 4 devices, each installed with Ubuntu 16.04, were assumed to act as ECUs, and automotive ethernet communication was facilitated using four RAD-Moon devices and one RAD-Pluto device. The configuration included one server ECU and four client ECUs, with each client assigned to a distinct domain. This experimental setup assumed the presence of four domains in total. A detailed description of the devices used is provided in Table 2.

**Table 2:** Description of the experimental setup.

Device	Description
Raspberry Pi 4	Assumed to act as an ECU within the vehicle, communicating with other Raspberry Pi devices using SOME/IP.
RAD-Moon	A media converter for connecting automotive ethernet devices, used to connect the Raspberry Pi 4 to the automotive ethernet.
RAD-Pluto	A switch for automotive ethernet, supporting four 100BASE-T1 ports, used to facilitate communication by connecting to the RAD-Moon.

In particular, this evaluation excluded centralized server-based methods, which are prone to single-point failures, and pairwise key-based methods, which are inefficient in publish/subscribe environments. Accordingly, the session establishment and communication overheads of the proposed protocol were compared with the group key-based method presented in [10]. To evaluate the performance of our proposed method and conduct a fair comparison with the approach, we utilized the open-source (https://github.com/netgroup-polito/secure-vsomeip) provided by [10]. This open-source is based on vsomeip version 2.10.21 and uses OpenSSL 1.1.1w for cryptographic operations (e.g., RSA-2048 and AES-256-GCM). In addition, we set the communication configuration to multicast mode for SOME/IP.

#### 5.2.2 Session establishment time.

**Fig 8:**
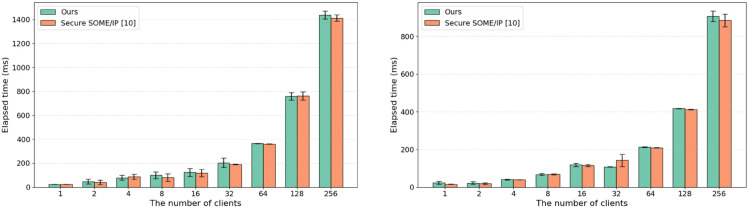
Session establishment time.

The time required to establish sessions in the proposed work was measured in both internal and external communication environments and compared with the existing study [10]. Note that internal communication means that both the server and clients are executed on one device, while external communication means that the server and clients are executed on two separate devices connected via automotive ethernet. In this experiment, up to 256 clients were created to evaluate the session establishment time, and each measurement was conducted 10 times for every client count. As shown in Fig 8, the session establishment time increased by approximately 6.80 ms for internal communication and approximately 6.26 ms for external communication compared to the existing study. This additional overhead is attributed to the domain ID comparison process introduced in our proposed method. Furthermore, the total elapsed time in the internal communication environment reached approximately 1,400 ms, whereas in the external environment, it was around 800 ms. This discrepancy is likely due to both the server and client processes running on the same hardware platform in the internal case, resulting in higher resource contention (e.g., CPU and memory usage).

#### 5.2.3 Communication overhead.

**Fig 9:**
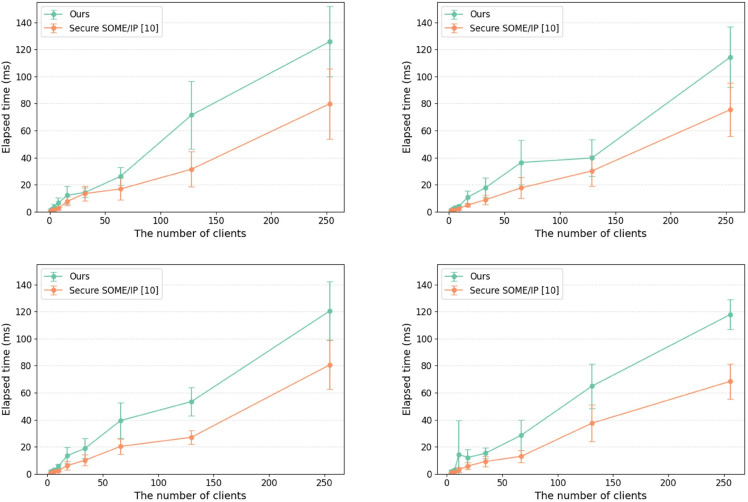
Communication overhead according to the number of domains.

**Fig 10:**
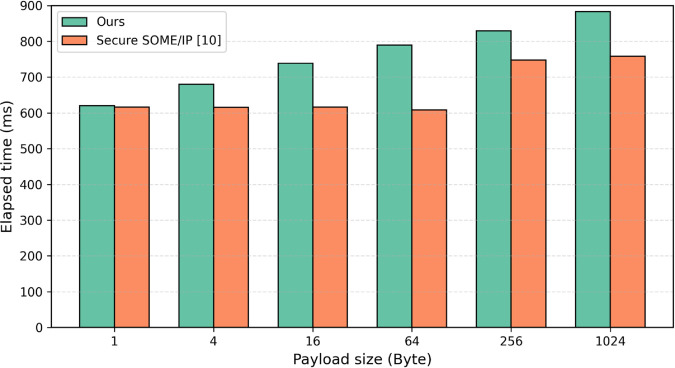
Communication overhead according to payload size.

We evaluated the communication overhead based on the number of domains and the payload size. For the communication overhead measurement based on the number of domains, we configured up to four domains and created up to 256 clients, as illustrated in Fig 9. Each measurement was repeated 10 times for every client count, and the payload size was set to 1,024 bytes. As the number of clients and domains increased, the communication overhead introduced by the proposed method was observed to be higher than that of the existing approach [10]. In particular, with 256 clients, the communication overhead increased by approximately 40 ms. To evaluate the communication overhead according to the payload size, messages with a maximum size of 1,024 bytes were transmitted in the environment with four domains, and the results are presented in Fig 10. When compared with the existing study [10], the message generation and transmission times of the proposed protocol were slightly higher. For a payload size of 1 byte, the existing study reported a message generation and transmission time of 616 ms, while the proposed protocol required 620 ms, showing almost no difference. However, as the payload size increased, the message generation and transmission times also increased slightly. Notably, for a payload size of 1,024 bytes, the existing study reported a time of 758 ms, while the proposed protocol required 883 ms, resulting in a difference of 125 ms.

This minor increase in communication overhead can be attributed to the fact that the existing study transmitted a single message without distinguishing between domains. In contrast, the domain key-based secure SOME/IP protocol proposed in this paper requires generating and transmitting separate messages for each domain, leading to the observed difference in message generation and transmission times.

#### 5.2.4 Domain attack experiment.

The MITM attack test was conducted to evaluate robustness against node compromise attacks. Assuming the presence of domains 1, 2, 3, and 4 and that the domain session key for domain 1 was leaked, the MITM attack results are summarized in Table 3.

**Table 3:** Comparison of MITM attacks (√: possible, *Χ*: not possible).

	Domain 1	Domain 2	Domain 3	Domain 4
Secure SOME/IP[10]	**√**	**√**	**√**	**√**
Ours	**√**	***Χ***	***Χ***	***Χ***

In the case of the existing study [10], which uses a single session key for all domains, it was observed that messages were successfully injected into all domains once the session key was leaked. In contrast, the security protocol proposed in this study successfully contained the attack to domain 1, where the domain session key was leaked. Message injection attacks targeting domains 2, 3, and 4 failed, demonstrating that the proposed protocol effectively restricted the attack to domain 1 only.

## 6 Discussion

### 6.1 Server ECU compromise

The domain-based secure SOME/IP protocol proposed in this paper assumes that the server holds all domain session keys and establishes sessions with all clients. If an attacker compromises a client ECU, the impact of the attack is confined to the domain that includes the compromised client. However, if the server ECU is compromised, the attacker can gain access to all domain session keys stored on the server, thereby jeopardizing all connected clients.

To detect and respond to cyber attacks on the server ECU, existing technologies such as Intrusion Detection Systems (IDS) [17] and network isolation techniques [18] should be employed. These methods enable a proactive response, even in the event of a server ECU compromise.

### 6.2 Key update

**Fig 11:**
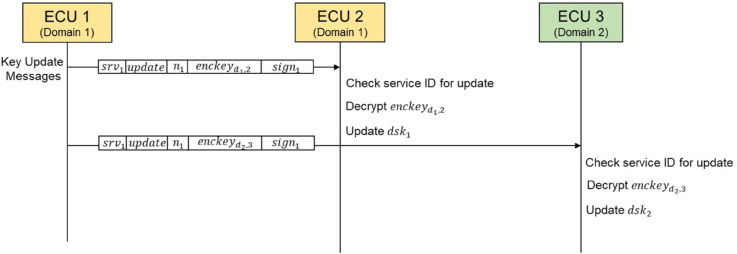
The key update process of the domain key-based secure SOME/IP protocol.

According to the work [19], the absence of a key update process poses potential risks as follows. First, if a new client subscribes and gains access to the domain session key, it could potentially access previously transmitted packets. Second, if a client unsubscribes and leaves the session but retains the domain session key, it could still transmit messages using the key, even without participating in the session. To prevent such issues, a key update process for the domain session key is necessary, which can be implemented using a public key-based method as shown in Fig 11. First, the server generates new domain session keys (${\text{dsk}}_{d_{i},i}$) and then sends key update messages containing a service ID, an update flag, a nonce, an encrypted domain session key (${\text{enckey}}_{d_{i},i}$), which is encrypted with each client’s public key (${\text{PK}}_{i}$), along with a signature, to each client. .Second, each client validates the service ID and the signature of the key update message. Then, it decrypts the encrypted domain session key (${\text{enckey}}_{d_{i},i}$) with its private key (${\text{SK}}_{i}$) and replaces its stored domain key. In addition, by assigning a life-cycle to each domain session key (${\text{dsk}}_{d_{i},i}$), the risk of key leakage and potential attacks can be effectively mitigated through periodic key replacement.

## 7 Conclusion

This paper proposes a domain key-based secure SOME/IP protocol. In the proposed method, domain session keys are exchanged between a server ECU and client ECUs using authorization information included in their certificates. Only authorized server and client ECUs can establish a session to exchange domain session keys. The server and client use the domain session key to encrypt/decrypt, and authenticate messages, ensuring that even if a node is compromised, the scope of the attack is limited to the domain of the compromised node.

Through experiments, the proposed method was compared with existing studies in terms of session establishment time, message transmission time, and robustness against node compromise attacks. With three domains, the proposed method showed an increase in session establishment time of approximately 5∼10 ms and a maximum increase in message transmission time of 115 ms compared to the existing group key-based approach. However, when an ECU was compromised, the proposed method restricted MITM attacks to the domain of the compromised ECU, whereas the group key-based approach allowed MITM attacks to affect all domains.

Future research aims to develop IDS and network isolation technologies to enhance the resilience of SOME/IP against server ECU compromise attacks.
